# Funding and Organization of US Federal Health Security Programs

**DOI:** 10.1089/hs.2016.0101

**Published:** 2017-02-01

**Authors:** Crystal R. Watson, Matthew Watson

Following the anthrax and 9/11 terrorist attacks of 2001, Presidents Bush and Obama both prioritized and supported investment in federal programs aimed at improving US defenses against health security threats, including major disasters; naturally occurring infectious disease epidemics; accidental releases of chemical, biological, or radiological materials; and chemical, biological, radiological, or nuclear (CBRN) terrorism.

Over the past 16 years, White House and Congressional leadership has jointly, and in a bipartisan way, established and supported federal programs to:

• Assess and understand natural, accidental, and intentional health security threats;
• Prevent CBRN weapons proliferation and establish norms against weapons use;
• Enable local public health and healthcare systems to manage major disasters and epidemics;
• Support research, development, procurement, and stockpiling of drugs, vaccines, and diagnostics that may be critical to saving lives in a national health emergency;
• Support planning for emergency response at the federal, state, and local levels;
• Address the overlap between human and animal infectious disease through a One Health initiative linking biosurveillance, planning, and response efforts; and
• Research and establish paths forward for national recovery from major health emergencies and catastrophic events.

Health security work in government is highly multidisciplinary, involving participation from experts in national security, public health, medicine, veterinary medicine, basic science, and international diplomacy, to name a few of the disciplines involved. As a result, programs to address health security are also spread among numerous government agencies, including:

• Department of Health and Human Services (HHS)
• Department of Homeland Security (DHS)
• Department of Defense (DoD)
• Department of State
• Environmental Protection Agency (EPA)
• Department of Agriculture (USDA)
• Department of Justice (DoJ)
• Department of Energy (DoE)
• Department of Commerce (DoC)
• National Science Foundation (NSF)

We estimate that funding for all federal programs focused on defense against threats to health security will total approximately $12.9 billion in fiscal year (FY) 2017.^1^ Of the amount budgeted for health security in the coming year, a majority of funding (over 50%) will be dedicated to programs that strengthen US preparedness and response broadly, by improving medical and public health infrastructure, developing countermeasures to address multiple threats and hazards, and preventing CBRN incidents from occurring in the first place. Approximately 20% of federal efforts and funding are directed toward addressing radiological and nuclear threats to the homeland, around 13% for biosecurity programs to address bioterrorism, 10% to address pandemic influenza and emerging infectious diseases, and approximately 3% for chemical security and defense.

While this overall funding number may seem large, it is important to note that these funds will be divided over the multiple programs and federal agencies mentioned above, and they are also essential for supporting state and local preparedness and response to health security events for the entire country and for surveillance and prevention internationally.

With the broadly distributed nature of these programs and associated funding streams, coordination of federal health security efforts is challenging. Currently, there is no systematic accounting by the federal government of those programs that are essential for building health security. Agency budgets do not easily identify these programs, and neither the Office of Management and Budget (OMB) nor Congress officially tracks funding and management of health security–related programs as a whole.

Because health security is so complex, multidisciplinary, and diffuse, the White House itself is best positioned to lead and coordinate this work as head of the executive branch. There are a number of steps that the new Administration can take to strengthen and coordinate the health security enterprise across agencies and programs.

## Recommendations

➢ **Establish a dedicated leader at the White House who is responsible for federal health security.**

Managing US health security programs requires high-level coordination, something that can be done best from the White House and through a dedicated leader focused exclusively on this important mission space. Past administrations have established similar leadership for related issues, such as biodefense, to provide a focal point for coordination of federal programs and policies, as well as a standing base from which to operate when a health security event like Ebola occurs, which is inevitable. The White House, therefore, should establish a dedicated leadership position and office to head federal health security efforts in the executive branch. This measure would greatly improve the Administration's ability to build health security and manage emergencies effectively.

➢ **Identify and account for health security programs across government.**

Creating a strong health security enterprise with programs that improve our national preparedness and response begins with the straightforward, but not simple, task of identifying the many programs and funding streams that contribute to building our defenses. While complete visibility is likely not possible, it is important for the White House to set strategic-level goals and priorities for health security programs, from which policies can be formed and programs informed. This can be done by both the executive agencies and the White House through the budgeting process. Agencies should be asked to identify those programs that contribute to health security and classify them as such in their annual budget-setting process. From that information, the OMB, as an office of the White House, can collect and define the universe of health security programs across agencies. This will enable better accounting and oversight, as well as ease of identifying gaps in our health security enterprise.

➢ **Stop reductions in health security funding and invest in systems-building.**

Support for the health security mission in the executive branch and by Congress through appropriations is critical. These are the programs that will enable recovery if a CBRN attack or pandemic occurs, will prepare us to respond effectively, and will save lives—potentially many thousands or even millions of them—if and when the US experiences such catastrophic health events. Yet, despite their recognized importance and bipartisan support, many health security programs have seen significant funding reductions in the past decade, which have in turn reduced our overall national preparedness to deal with catastrophic health events. In FY2017 alone, funds are estimated to decrease by almost $800 million from the prior year.

This is an area in which we cannot afford to be complacent and should not take our past work and success for granted. Health security is a national security imperative and should be treated and funded as such by the new Administration.
